# Therapeutic Benefits of Selenium in Hematological Malignancies

**DOI:** 10.3390/ijms23147972

**Published:** 2022-07-19

**Authors:** Melanie A. Ehudin, Upendarrao Golla, Devnah Trivedi, Shobha D. Potlakayala, Sairam V. Rudrabhatla, Dhimant Desai, Sinisa Dovat, David Claxton, Arati Sharma

**Affiliations:** 1 Division of Hematology and Oncology, Department of Pediatrics, Pennsylvania State University College of Medicine, Hershey, PA 17033, USA; ehudinmelanie@gmail.com (M.A.E.); sdovat@pennstatehealth.psu.edu (S.D.); 2 Division of Hematology and Oncology, Department of Medicine, Pennsylvania State University College of Medicine, Hershey, PA 17033, USA; ugolla@pennstatehealth.psu.edu (U.G.); dclaxton@pennstatehealth.psu.edu (D.C.); 3 Penn State Cancer Institute, Pennsylvania State University College of Medicine, Hershey, PA 17033, USA; tdevnah@gmail.com (D.T.); ddesai@pennstatehealth.psu.edu (D.D.); 4 Department of Biological Sciences, School of Science Engineering and Technology, Penn State Harrisburg, Middletown, PA 17057, USA; sdp13@psu.edu (S.D.P.); svr11@psu.edu (S.V.R.); 5 Department of Pharmacology, Pennsylvania State University College of Medicine, Hershey, PA 17033, USA

**Keywords:** selenium, selenoprotein, cancer, chemoprevention, chemotherapeutic, radiotherapy, leukemia, hematologic malignancies, murine disease model

## Abstract

Supplementing chemotherapy and radiotherapy with selenium has been shown to have benefits against various cancers. This approach has also been shown to alleviate the side effects associated with standard cancer therapies and improve the quality of life in patients. In addition, selenium levels in patients have been correlated with various cancers and have served as a diagnostic marker to track the efficiency of treatments or to determine whether these selenium levels cause or are a result of the disease. This concise review presents a survey of the selenium-based literature, with a focus on hematological malignancies, to demonstrate the significant impact of selenium in different cancers. The anti-cancer mechanisms and signaling pathways regulated by selenium, which impart its efficacious properties, are discussed. An outlook into the relationship between selenium and cancer is highlighted to guide future cancer therapy development.

## 1. Introduction

Due to the high incidence rate of individuals diagnosed with cancers and the corresponding harmful effects to their quality of life from treatment, determined efforts have focused on improving cancer prevention and treatment, which have been pursued throughout the literature [1,2,3,4]. Cancer is a disease characterized by normal cells mutating and proliferating uncontrollably and/or by the improper gene expression or signaling pathways of specific targets, which results in malignant transformations [5,6]. Typically, chemotherapy, immunotherapy, radiation therapy, or surgery is employed to treat cancer patients [3,7]. The earlier the cancer is detected, staged, and aggressively treated, the better the prognosis of the disease, and this may also prevent cancer from metastasizing to other parts of the body [7]. Blood cancers, such as acute myeloid leukemia (AML) and acute lymphoblastic leukemia (ALL), are characterized by the over-proliferation of blasts in the blood, bone marrow, and other tissues, which can hinder the hematopoietic pathways. AML is a rapidly progressive cancer that is heterogenous in nature, making it difficult to effectively treat; therefore, it is associated with low survival and high relapse rates. This is especially true for the elderly, who commonly exhibit pre-existing conditions that can be compounded by side effects from the treatment or the disease itself [8]. Among the cancers that children are diagnosed with, leukemia is one of the most prevalent, wherein acute lymphoblastic leukemia (ALL) is responsible for about one fourth of all childhood malignancies [9].

Further, among adults that are diagnosed with acute leukemia, AML is one of the most common forms and has a five-year survival rate of 26.9% [10]. The standard treatment for AML includes treating patients in the acute phase with high-dose induction chemotherapy to reduce the leukemic blast growth and with consolidation chemotherapy and/or allogenic stem cell transplantation in the remission phase [4,11]. The major drawbacks of current cancer therapy include a lack of selectivity between targeting cancer versus healthy cells (which can lead to severe infections), the toxicity associated with most current methods, and the development of drug resistance with continued treatment over time [3,7]. Recent efforts have been focused on developing more effective and less toxic cancer therapies [12]. Reduced levels of selenium (Se) in the blood serum of children with AML (76.46 ± 24.59 μg/L) versus healthy children (102.38 ± 19.25 μg/L) before chemotherapy has been reported [13]. A trend of significantly reduced levels of serum selenium was also observed among fifty patients, 16 to 75 years of age, which were diagnosed with either ALL or AML but never previously treated, in comparison to healthy subjects [14]. Additionally, a significant difference among the serum selenium levels was shown when patients with different subsets of AML were compared [14]. Investigations have been driven to determine whether selenium deficiency causes the disease or is a resulting effect of cancer [13].

**Figure 1:**
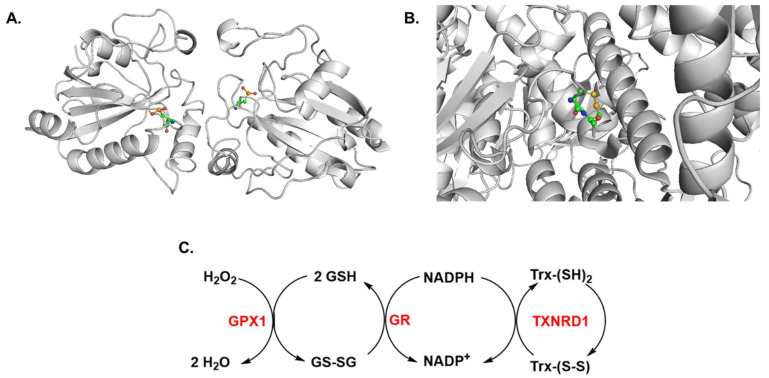
Selenium is required to ensure the expression and functionality of selenoproteins to protect cells from oxidative damage. (**A**) Structure of selenoenzyme glutathione peroxidase *GPX1* in *Bos taurus* (Bovine), which plays an essential role in detoxifying hydrogen peroxide (PDB: 1GP1). Selenium is a crucial intracellular part of this enzyme’s ability to protect cells. (**B**) Crystal structure of recombinant rat thioredoxin reductase *TXNRD1* with oxidized C-terminal tail depicting the selenocysteine residue that is key for its catalytic activity (PDB: 3EAO). The functionally important selenocysteine residue present in the active site of (**A**,**B**) is highlighted in color. (**C**) Scavenging of ROS by *GPX1* and catalytic redox cycle of selenoprotein thioredoxin (Trx) by Trx reductase *TXNRD1*. GR, glutathione reductase; GSH, glutathione (reduced form); GSSG, glutathione disulfide.

Selenium is an essential, naturally occurring trace mineral element, implicated in a diverse set of biological processes that impact health and disease [15,16,17,18,19,20,21]. Among the three elements (oxygen, sulfur, and selenium) from the chalcogen group of the periodic table that are incorporated into biological macromolecules, both sulfur and Se exhibit similar chemical characteristics in terms of valence states (−2, 0, +2, +4, and +6). Although the two elements are utilized in a variety of biochemical reactions, selenium is found to be more abundant in biological systems than sulfur due to its stability, reactivity, and the greater polarity of its chemical bonding [22,23,24]. Selenium is a key component in several metabolic pathways, including the antioxidant system, DNA stability and the production of proteins and nucleic acids, redox signaling, thyroid hormone metabolism, and the immune system [18,19]. The amino acid selenocysteine (Sec) is present in the active site of 25 selenoproteins in humans (e.g., glutathione peroxidase (GPX) or thioredoxin reductase (TXNRD), Figure 1) and selenoproteins require selenium to function properly [15,25]. The selenium-based enzymes such as GPX and TXNRD (Figure 1) have important roles in multiple biological pathways and are utilized by cells to protect against oxidative damage on the cell membrane and in DNA, which may prevent or protect against cancer [26,27]. Although the majority of studies have highlighted the chemopreventive role of selenium, recent randomized control trials and observational studies have reported that selenium alone failed to exhibit anticancer activity [28,29,30]. Conversely, selenium supplementation has raised several concerns for increased risks of cancer and detrimental effects, especially in conjunction with type 2 diabetes [31]. The oxidation of NADPH to NADP^+^ by enzymes such as thioredoxin reductases (Figure 1) is necessary for the reduction of reactive oxygens species (ROS) such as hydrogen peroxide (H_2_O_2_) to water by glutathione peroxidases. These GPXs significantly contribute to protecting cells against oxidative damage from ROS and reactive nitrogen species (RNS) including superoxide, H_2_O_2_, and nitric oxide (NO) [25,32]. Selenium supplementation modulates the NO-mediated apoptosis induced by cadmium [33]. A deficiency in selenium results in the increased expression of inducible nitric oxide synthase (iNOS) and NO production [34]. The crystal structures of the selenoproteins in Figure 1 were obtained from the Protein Data Bank (PDB) and generated in PyMOL [35]. The significant role of selenium in major biological processes and activities suggests the potential therapeutic benefit that may come from incorporating it into standard-of-care cancer therapies. Herein, we present a detailed survey of the literature that demonstrates the anti-cancer activities exerted by selenium. The mechanisms or pathways in which selenium imparts its activity and its potential towards treating different cancers will be discussed, with a focus on hematological cancers. The compatibility of selenium supplementation alongside conventional cancer treatments such as chemotherapy and radiotherapy will also be examined. These insights will aid future investigations with the aim of improving the efficacy and minimizing the toxicity of cancer therapies.

## 2. Regulation of Signaling Pathways by Selenium in Cancer

### 2.1. Selenoproteins Play a Role in Cancer

Selenium is present in all mammals and is utilized by selenoproteins (Figure 1), a family of proteins that need the selenium containing amino acid, selenocysteine, to effectively carry out target processes [36,37]. Selenium has its own mRNA codon that allows for its insertion as selenocysteine into selenoproteins [38]. Selenoproteins play a crucial role in protecting cells from cancer. Thus, a deficit of selenium in the diet could result in the depletion of selenoprotein levels and consequently deprive the body of their benefits [38,39]. Selenoproteins also have roles in DNA-repair and cytokine control pathways [40,41]. Differential expression profiles of GPXs and TXNRDs indicate that selenoprotein families have importance in carcinogenesis, the occurrence of cancer, and influence the immune-cell subtypes, mechanistic cell infiltration, and tumor cell stemness [38,39]. These genes were found to have key roles in cancer survival (e.g., *TXNRD1* and *TXNRD3* are correlated with a poor prognosis), the tumor microenvironment (e.g., TXNRD1, GPX1, and *GPX2* are linked to tumor mutagenesis and development), and drug sensitivity (*TXNRD1*, *GPX1*, *GPX2*, and *GPX3* are associated with the formation of drug resistance) [26]. A low expression of *GPX3* has been correlated with the poor prognosis (overall survival and disease-free survival) of AML patients and suggests that targeting substrates related to the glutathione metabolic pathway may advance AML treatment [42]. The antioxidant thioredoxin system has also been reported to be upregulated in cancer cells and linked to cancer development, relapse, and chemoresistance in acute leukemia [43,44]. The ROS-facilitated signaling of c-Jun activation domain-binding protein-1 and thioredoxin have been correlated with the pathology, poor survival, and relapse in AML-M5 patients [45]. Recent findings indicate that single nucleotide polymorphisms (SNPs) in selenoprotein and selenium metabolic pathway genes alone or in combination with suboptimal levels of selenium contribute to cancer development [46]. Research and development considering the link between selenoproteins and tumorigenesis may allow for the design of more effective targeted therapies [26,43].

### 2.2. Role of Selenoproteins in Hematological Malignancies

Selenoproteins play an important biological role in maintaining human health by regulating selenium transport, redox homeostasis, thyroid hormone metabolism, and immunity, as stated above. The dysregulation of selenoproteins and selenium deficiency can result in several serious disorders such as cancer, cardiovascular disease (Keshan disease), osteoarthritis, liver disease (hepatopathy), arthropathy (Kashin–Beck disease), and a defective immunity against viral infections [25,47,48]. The dysregulation of the cellular redox systems plays an important role in both acute and chronic hematological malignancies [49]. The ubiquitous loss of mitochondrial thioredoxin reductase (TrxR2) in mice was associated with embryonic death at embryonic day 13 and the presentation of smaller embryos that were severely anemic with increased apoptosis in the liver [50]. A pan-cancer expression analysis revealed the association of selenoprotein P (*SELENOP*) with a better prognosis in most cancers, but a poorer prognosis in brain glioma and uterine corpus endometrioid carcinoma [51]. Furthermore, the overexpression of Glutathione peroxidases (GPXs) in AML patients compared to normal controls was significantly associated with a poor prognosis of overall survival [52]. The overexpression of the selenoprotein Glutathione peroxidase 4 (*GPX4*) contributed to the poor prognosis of aggressive diffuse large B-cell (DLBC) lymphoma via the inhibition of ROS-induced cell death [53]. Further, the downstream regulator of *GPX4*, i.e., *SECISBP2* (Selenocysteine Insertion Sequence-Binding Protein 2), which regulates various selenoproteins, was revealed as a novel prognostic predictor of DLBC lymphomas and may serve as a potential therapeutic target [54]. Recently, Eagle K et al. integrated pan-cancer genetic dependency data with that of a comprehensive enhancer landscape and identified *SEPHS2*, a selenoprotein biosynthesis gene, as a highly AML-selective dependency encoded by a Myb-regulated oncogenic enhancer. The suppression of the *SEPHS2*-regulated production of selenoproteins by diet selectively renders AML susceptible to oxidative stress without affecting normal hematopoiesis [55]. Overall, the current literature highlights the potential role of selenoproteins and selenium metabolism in different cancers including hematopoietic malignancies.

### 2.3. Inorganic and Organic Selenium Compounds Exert Therapeutic Activities

**Figure 2:**
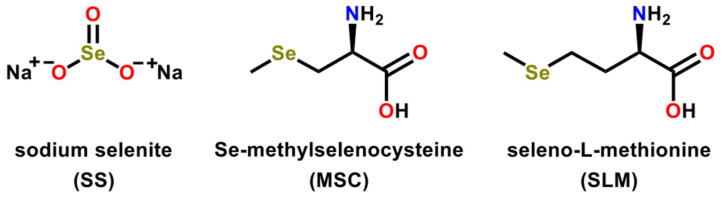
Chemical structures of sodium selenite (SS), methylselenocysteine (MSC), and seleno-L-methionine (SLM), which are well studied selenocompounds that have demonstrated anti-cancer properties.

**Figure 3:**
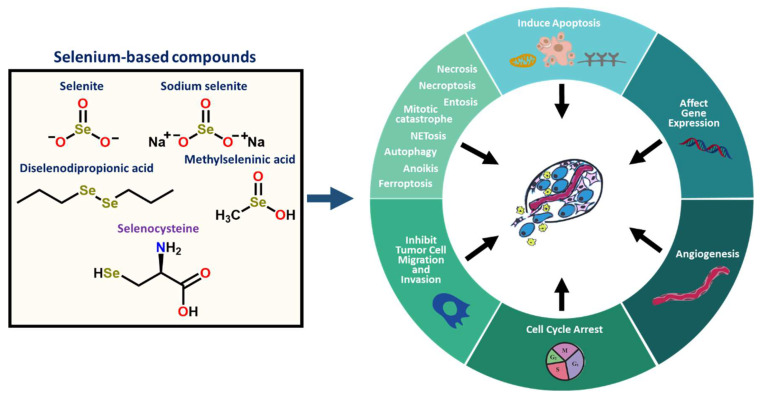
Selenium-based compounds exhibit chemopreventive or chemotherapeutic properties through regulation of various processes such as cell cycle arrest, apoptosis, angiogenesis, etc.

Multiple reports have shown that selenium in varying forms (e.g., organic, inorganic, or as selenoproteins) can exert anticarcinogenic or antimutagenic properties that allow them to serve as promising candidates for anticancer therapies (Figure 2 and Figure 3) [16,32,56]. It has been reported that Se possesses anti-proliferative, anti-inflammatory, and anti-viral activities in addition to immune altering properties and has been implicated in various cancers [56,57]. Inorganic selenium-based compounds (such as selenite and selenate) tend to metabolize into hydrogen selenide and organic forms of selenium (such as diselenides, selenides, selenoesters, methylseleninic acid (MSA), 1,2-benzisoselenazole-3[2H]-one and selenophene-based derivatives, and selenoamino acids and Selol) metabolize into methylselenol [57,58]. These highly reactive, redox-active metabolites (e.g., hydrogen selenide or methylselenol) exhibit distinct cellular functions and toxicities that give rise to their chemopreventive or chemotherapeutic properties [57,58,59].

Previous reports have focused on investigating the properties imparted by the organic selenocompounds such as methylselenocysteine (MSC) and seleno-L-methionine (SLM) (Figure 2) [57,60]. These studies have shown that, at supra-nutritional concentrations, MSC and SLM exhibit anti-angiogenic and anti-cancer effects. The well-studied inorganic selenium compound selenite (Figure 2) is clinically utilized and supplemented in the diet of patients with the endemic Keshan disease [61,62]. Keshan disease arises due to insufficient dietary selenium levels, which can result in cardiovascular atrophy if not properly addressed [61,62]. The supplementation of selenite into the diet successfully treats Keshan disease and the associated symptoms, suggesting the therapeutic benefits that can be imparted by selenite [61,62].

Several studies indicate the ability of selenocompounds to work well with conventional cancer therapies (e.g., chemotherapy or radiotherapy) to improve their efficacy toward malignant cells and decrease their associated off-target or adverse effects [63]. Multiple human studies [64,65,66] have demonstrated a decrease in the toxicity of the standard-of-care chemotherapies (e.g., cisplatin, doxorubicin, cyclophosphamide, and busulfan), and noted that the therapeutic benefit was not adversely affected upon supplementing cancer therapy with selenium either in the form of sodium selenite (SS) or the organic SLM (Figure 2). Xenograft studies have shown that SLM was more efficacious in improving conventional chemotherapies relative to SS [67,68]. The ability of organic Se-methylselenocysteine (MSC) versus SS and SLM (Figure 2) to efficiently produce methylselenol, a metabolite that has been suggested to regulate a large part of the interactions with standard chemotherapies, suggests that MSC is more promising and beneficial than SS and SLM [69,70]. Notably, the anticancer activity of MSC and SLM partly depends on the endogenous expression levels of various enzymes such as kynurenine aminotransferases (KATs) and cystathionine γ-lyase (γ-cystathionase) in target tissues along with their ability to synthesize chemopreventive metabolites, either methylselenol and/or seleno-keto acid metabolites, in situ [71]. SS is still preferentially studied by some labs because of its demonstrated increased selectivity for exerting a ROS-influenced cytotoxic effect that reduces the cell proliferation in malignant cells when compared to normal cells [72,73]. However, the greater genotoxicity induced by inorganic SS relative to SLM or MSC could result in possible late toxicities (e.g., myelodysplasia or acute leukemia), an important factor when selecting an agent to work beneficially with DNA-damaging cancer treatments [74].

### 2.4. Selenium-Based Compounds or Proteins Act by Various Modes of Action

Reports have indicated that selenium-based compounds or selenoproteins exhibit chemotherapeutic activity by regulating various biochemical pathways related to apoptosis [1], cell proliferation [56,75], cell cycle arrest [76,77], necrosis, autophagy, ferroptosis, necroptosis, entosis, anoikis, NETosis, or mitotic catastrophe imparting cytotoxic effects or cell death (Figure 3) [38,78]. The selenocompounds with promising anticancer activity were summarized in Table 1. The selenoprotein glutathione peroxidase 4 (*GPX4*) and glutathione can regulate ferroptosis to protect the cell from oxidative damage (e.g., excessive ROS/RNS production) [79,80]. However, the overexpression of *GPX4* has been correlated with the poor prognosis and overall survival of diffuse large B-cell lymphoma [54]. Advantageous responses from selenium-related cancer treatments have also been shown to be imparted by protein modification, the impairment of tumor angiogenesis, and the regulation of processes associated with DNA repair/damage, metastasis, or the endoplasmic reticulum (ER) and oxidative stress responses (Table 1; Figure 3). Selenium has been linked to metastasis in various cancers and is thought to impart anti-metastatic properties (Figure 3), such as reducing the expression of Osteopontin and suppressing cell motility, migration, invasion, and angiogenesis [78,81]. Selenocompounds have also been reported to increase the activity of macrophages and enhance cell respiration [13].

The amount of the selenium agent administered can influence whether prooxidant or antioxidant activity is observed. Typically, nutritional doses exert antioxidant and chemopreventive effects relative to supranutritional concentrations that can induce prooxidant and anticancer pathways [82]. The metabolites derived from selenium have been linked to the triggering of oxidative stress in cells via the production of ROS/RNS, which in turn leads to the oxidation of protein thiol moieties [32,56]. Selenium-based compounds have been shown to exhibit chemopreventive and anticancer properties through prooxidant activities and the regulation of cellular redox homeostasis by altering thiol groups in multiple metabolic pathways, stimulating the production of ROS/RNS, and regulating changes in the chromatin [57,83].

Selenium-based compounds have been shown to modulate cellular responses by regulating p53 phosphorylation [76,84,85,86]. Selenium treatments have been demonstrated to increase the host cell’s reactivation of a UV-damaged reporter plasmid template [87], indicating its possible role in DNA repair [86,88]. However, it has been established that selenium can only regulate DNA repair in wildtype p53 containing cells [86,88]. The selenoprotein thioredoxin reductase and redox factor 1 (*Ref-1*) are needed for p53 cysteine reduction, which allows for the prevention of DNA damage [88,89,90]. Studies have shown that SLM activates DNA repair and shields cells from DNA damage without inducing cell cycle arrest or apoptosis [76,84,85,86,88]. In one study, mouse embryonic fibroblasts, either wildtype or null for p53 genes, were pre-treated with the nontoxic agent, SLM [86]. This pretreatment induced a DNA repair response and protected the fibroblasts against DNA damage in the presence of UV radiation or UV-mimetic chemotherapy [86]. The beneficial activity imparted by SLM was not seen in cell lines that were null for the tumor suppressor p53 gene. Therefore, p53 may be crucial for distinguishing healthy and cancer cells [86]. On a similar note, selenium nanoparticles exhibited an inhibitory effect on p53’s ability to mitigate chemotherapy-induced diarrhea and possibly serve as potential chemoprotectants [91].

The addition of Se alongside treatment with DNA-damaging chemotherapeutics has been shown to selectively protect healthy tissues and prevent the typically observed dose-limiting toxicity [86]. This has allowed for the administration of increased chemotherapeutic doses and has induced a cytotoxic effect on cancer cells [86]. Human tumor xenograft murine models (human squamous cell carcinoma of the head and neck, FaDu and A253, and colon carcinomas HCT-8 and HT-29) that were administered seleno-L-methionine prior to and during chemotherapy treatment experienced an improved tolerance to higher doses of irinotecan [92]. The ability to treat tumor-bearing mice with higher concentrations of drugs allowed for an enhanced efficacy towards chemoresistant tumors [92]. Phase 1 trials have explored the potential of SLM to improve chemotherapy and found that patients could tolerate high doses of SLM without displaying adverse toxic effects [93,94].

Selenium-derived compounds have been shown to alter the expression of various target genes or modulate the interplay between signaling networks [83,114]. Powers and co-workers demonstrated that selenium supplementation in the form of selenite plays a role in cellular signaling and its consequences by regulating the highly conserved Delta–Notch signaling pathway [61]. In vitro studies indicated that selenite treatment on primary mouse hepatocytes, MCF7 breast adenocarcinoma cells, or HEPF2 liver carcinoma cells altered the transcription levels of various genes involved in the Delta–Notch signaling network (e.g., Notch1) [61]. The intraperitoneal administration of selenite (2.5 mg/kg) to mice resulted in significantly reduced Notch1 expression levels in liver and kidney tissues [61]. These results provide support for selenite’s role as an inhibitor in the Notch signaling pathway and its promise as a targeted therapy of Notch, which has been correlated with the prognosis in various cancers, fibrosis, and neurodegenerative diseases [61,115].

## 3. Role of Selenium and Its Therapeutic Advantages in Various Cancers

Selenium has been linked to the pathophysiology of a diverse set of conditions (e.g., depression, atherosclerosis, and cancer) [15]. In 1969, populations with an increased selenium intake were found to have a decreased cancer incidence and thus paved the way for research into the therapeutic benefit of selenium [116,117]. Additional evidence suggested that selenium could be useful in cancer treatment and prevention [56,118,119]. Studies have also shown a link between cancer progression, incidence, or overall age-adjusted cancer mortality and selenium intake at supranutritional doses or the level of selenium in the serum [13,120,121].

### 3.1. Pre-Clinical Selenium-Based Therapeutic Studies Are Promising in Disease Animal Models

Animal studies have shown the chemotherapeutic properties imparted by selenium in different types of cancer [122,123,124]. Many murine or rat-related studies that investigate the effect of selenium in cancer models have provided selenium in the rats’ food or drinking water [125]. Upon supplementing the diet with 5 ppm of selenite, the rats with liver tumors that were induced by the azo dye (*m′*-methyl *p*-dimethylaminoazobenzene) experienced a fifty percent reduction in tumor development [126]. Mammary tumorigenesis rat models that were fed with a selenium supplemented diet, in the form of SS, showed that tumorigenesis was inhibited [125]. Medina and Shepherd investigated the tumor-inhibiting efficacy of dietary selenium on the primary hyperplastic alveolar nodules (HAN) of the mammary glands of BALB/c mice [127]. They observed a significant reduction in the tumor-producing capability of hyperplastic alveolar nodules and the inhibition of mammary tumorigenesis when the mice were fed dietary selenium. These results suggest that selenium could be a promising candidate to inhibit neoplastic transformation in preneoplastic cells and to hinder their formation [127]. Selenium has also been reported to effectively exert anti-cancer effects associated with its oxidative activities in bone cancers without adversely affecting healthy tissues [128].

Jiang and co-workers revealed that chitosan oligosaccharide-conjugated selenium (COS-Se) was selectively active in human gastric cancer (SGC-7901) versus normal fibroblast (L-929) cells [129]. The potency of COS-Se in human gastric cancer cells was exemplified through enhanced immune function activities and reduced cancer growth, proliferation, and metastasis. This COS-Se was well tolerated and presented no toxicity in mice. Gastric adenocarcinoma SGC-7901 murine models that were subcutaneously injected and orally administered with COS-Se for 28 days exhibited significantly reduced tumor volumes and weights, reduced expressions of pro-angiogenesis related factors (e.g., CD34 and vascular endothelial growth factor (VEGF)) in the treated tumor tissues, and decreased serum levels of tumor metastasis-related targets (e.g., matrix metalloproteinase-9 (MMP-9) and VEGF) relative to the control group, demonstrating the anti-cancer efficacy of COS-Se in vivo. These findings suggest that dietary supplementation with selenium-oligosaccharides may be promising in cancer prevention and may play a critical role in improving gastric cancer disease [129].

Karelia et al. reported the potency of a novel selenazolidine-bis-aspirinyl derivative, AS-10, in pancreatic ductal adenocarcinoma (PDAC) to overcome the poor survival rates and negative effects of standard PDAC therapeutics [130]. The PDAC cell lines were sensitive towards AS-10, exerting a selective cytotoxic effect, wherein the cell proliferation of the cancer cells was reduced relative to non-malignant mouse embryonic fibroblasts that were unaffected at similar concentrations of AS-10. The treatment of the PDAC cell line, Panc-1, with AS-10 exhibited caspase and ROS-mediated apoptosis without activating necrosis or DNA damage genes, reduced nuclear transcription factor-kappa B (NF-κB) signaling by inhibiting the proinflammatory cytokine TNF-α, and arrested G_1_/G_0_ cell cycle progression. The observations from in vitro studies were supported by RNA-sequencing and bioinformatic analysis, which indicated that AS-10 played a role in NF-κB signaling and pathways related to the cell cycle, death, and survival, while not affecting DNA damage marker genes. The cytotoxic and pro-apoptotic properties of AS-10 in PDAC cell lines and the synergistic effects observed upon treating PDAC cells with a combination of gemcitabine and AS-10 suggest the potential for selenium-based compounds to treat patients diagnosed with PDAC [130].

Numerous studies have reported potential therapeutic benefits from selenium supplementation or the use of selenium-derived compounds in breast cancers [131,132,133,134]. There are limited available treatments for triple-negative breast cancer (TNBC) patients, especially since these types of patients tend to develop chemoresistance [135]. This subtype of breast cancer is aggressive, tends to metastasize, and results in poor survival [135,136]. Patients with TNBC have been demonstrated to exhibit increased levels of VEGF, a tumor angiogenesis and vascular permeability inducer, resulting in cancer stem cell formation and tumor proliferation, migration, and survival [137,138,139]. Antiangiogenic treatments such as the monoclonal antibody against VEGF (Avastin, also known as bevacizumab) have been shown to be therapeutically beneficial towards treating TNBC patients; however, at high doses, these agents can have adverse effects [137,140]. Antiangiogenic drugs have been shown to induce distant metastasis and result in the poor prognosis of TNBC patients [137,141].

**Figure 4:**
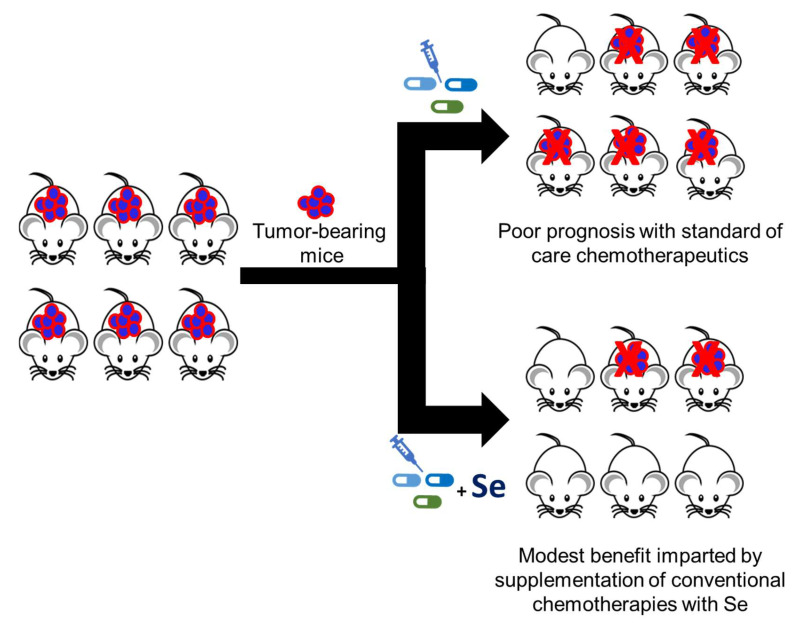
Standard cancer therapy administered in conjunction with selenium may exhibit improved efficacy in tumor-bearing murine models.

Nutritional supplements such as marine-based fish oil and selenium have been shown to have chemotherapeutic properties [142,143]. The potency of selenium in an in vitro model of lung adenocarcinoma was increased with the addition of fish oil [142]. Reduced concentrations of selenium in the blood of breast cancer patients [144] and the established anticancer properties of selenium (e.g., exerting oxidative stress in tumor tissues, reducing tumor growth and metastasis, decreasing VEGF expression, enhancing immune resistance, and diminishing antitumor drug resistance) suggest that selenium could be an apt treatment for TNBC patients [137,145]. Peng et al. [137] found that the addition of fish oil and selenium augmented the efficacy of the low-dose antiangiogenic antibody Avastin on triple-negative 4T1 mammary-carcinoma mice (Figure 4). The mice exhibited reduced tumor progression and metastasis with the combination treatment relative to Avastin alone (Figure 4) [137]. The observed increase in the antibody’s efficacy with the combination-treated mice was attributed to the regulation of multiple signaling pathways by examining the mRNA and protein expression of cancer-related signaling molecules [137]. The combination treatment of Avastin, fish oil, and selenium reduced the expression of various potent proangiogenic (growth) factors and their membrane receptors, inhibited downstream targets (e.g., transcriptional factors, kinases, and cancer stem cell markers), and induced a pro-apoptotic tumor effect, thereby supporting the potential therapeutic value of selenium in breast cancer patients [137].

Li and coworkers [146] observed synergistic effects, such as a reduction in tumor growth, upon treating MCF-7 breast cancer xenograft models in ovariectomized female athymic nude mice, with a combination of tamoxifen and MSC relative to a single drug treatment (Figure 4). The immunohistochemistry and TUNEL analysis indicated that MSC or combination treatment significantly lowered ERα expression and ERα-related genes (e.g., progesterone receptor and cyclin D1 expression), the Ki-67 index, and the microvessel density, and enhanced the percentage of apoptosis in tumor tissues. The efficacy imparted by the organic selenium compound MSC and tamoxifen on ERα-positive breast cancer mouse xenografts models (Figure 4) demonstrates the compatibility of selenium working in conjunction with conventional cancer therapies [146]. It also suggests the therapeutic potential of selenium as an adjuvant therapy or in chemoprevention.

### 3.2. Clinical Studies Support Adjuvant Selenium Supplementation

Clinical trials have demonstrated the efficacy that can be imparted by selenium supplementation during chemotherapy in the development of various cancers such as prostate, lung, and colorectal cancer [118,147,148]. In clinical trials, it has been observed that selenium and vitamin C supplementation decrease the incidence and mortality of gastric and lung cancer [149,150]. Patients with a past medical record of non-melanoma skin cancer that took 200 μg daily of elemental Se, as selenized yeast, had a decreased incidence of colon, lung, and prostate cancers in a Nutritional Prevention of Cancer (NPC) study, which supported the potential of selenium towards chemoprevention [69].

Patients with lung and breast cancer that were being treated with cisplatin experienced significantly improved peripheral white blood cell counts when they also received 400 μg per day of selenium in the form of Seleno-Kappacarrageenan as compared to patients that underwent a regimen without selenium supplementation (3.35 ± 2.01 versus 2.31 ± 1.38 [×10^9^ L])/L, *p* < 0.05) [151]. Selenium supplementation in the form of Seleno-Kappacarrageenan increased serum selenium levels from 70.4 ± 22.86 to 157.04 ± 60.23 ng/mL (*p* < 0.001), reduced the consumption of the granulocyte colony-stimulating factor, and decreased volumes of blood transfusion without systemic toxicity [86,151]. It was also shown in this study that selenium could potentially be utilized to reduce the nephrotoxicity and bone marrow suppression that is exhibited with the conventional drug treatment, cisplatin [151].

Supplementing chemotherapy with selenium also had efficacious results for ovarian cancer patients [152]. Patients that were undergoing Protecton**^®^** Zellactiv (Smith Kline Beecham, Fink Naturarznei GmbH, Bühl, Germany) chemotherapy and that ingested selenium daily experienced significantly higher levels of serum selenium, enhanced glutathione peroxidase 1 (*GPX1*) activity in their erythrocytes, higher levels of malondialdehyde (MDA), increased white blood cells counts, reduced neutropenia, and a reduction in the negative side effects induced by chemotherapy (e.g., hair loss, abdominal pain, weakness, malaise, and loss of appetite) [152]. A selenium supplementation did not negatively impact the efficacy of chemotherapy in the studies carried out by Hu et al. [151] and Sieja et al. [152]. In a retrospective study, Meyer et al. examined the serum in renal cell cancer patients and their survival [153]. The researchers reported that reduced levels of serum selenium and selenoprotein P, the major selenium transport protein in blood, upon diagnosis were correlated with a poor, five-year survival [153]. These clinical findings suggest that adjuvant selenium supplementation may allow conventional therapy to be more effective in selenium deficient patients diagnosed with various cancers and suggest that monitoring selenium levels in these types of patients could be a key biomarker to track the efficiency of treatments or to gain further insights into the disease.

## 4. Selenium Reduces Disease Progression in Blood Cancers

Selenoproteins have been reported to have crucial roles in the immune system pathways regulating T-cell proliferation, differentiation, and metabolism signaling that can have implications in various diseases [154]. Selenium supplementation has been shown to have anti-cancer properties corresponding to a reduced cancer cell survival and to activate antibody formation and the activity of helper T-cells, cytotoxic T-cells, and natural killer cells [14,154,155]. The fundamentally important element, Selenium, can be found in red blood cells and in blood proteins such as hemoglobin and albumin [156,157]. Acute leukemias have been associated with the modified bioavailability and/or altered levels of trace elements by reducing absorption, enhancing urinary loss, and modifying element-binding proteins [14,155].

### 4.1. Selenium Induces a Cytotoxic Effect in Leukemia/Lymphoma Cells

Multiple studies have shown the chemotherapeutic properties of selenocompounds in different leukemia/lymphoma cells (e.g., the AML HL-60 cell line) and their ability to induce cytotoxic effects or protective activities to prevent healthy tissue toxicities without compromising the treatment’s efficiency [158,159,160,161,162]. MSA has also been reported to sensitize human B-cell acute lymphoblastic leukemia (B-ALL) cell lines to standard chemotherapeutics, wherein cytotoxic activities were increased with the addition of MSA [160]. The synergistic effects of MSA were in part attributed to inhibiting the NF-κB pathway [160]. Wu and co-workers demonstrated that the treatment of human T-cell acute lymphoblastic leukemia (T-ALL) cell lines, Jurkat, and MOLT-4 with a selenium–platinum compound reduced cell proliferation relative to the platinum-only analogue, induced cell cycle arrest, and exerted caspase and ROS-mediated pro-apoptotic activities by regulating a mitochondrial signaling network [163]. Additionally, the well-studied SS exerted a cytotoxic and pro-apoptotic effect on the human T-ALL cell line, MOLT-3, resulting in a reduced cellular proliferation in a dose-dependent manner [164]. Mouse leukemia cells such as L1210, L797, and L615 were also shown to be sensitive to SS via cytotoxic assays [165,166]. The selenite treatment of mouse leukemic L1210 cells induced DNA single-strand breaks and a pro-apoptotic effect, and stimulated endonucleases that resulted in DNA double-strand breaks [165]. In addition, an in vivo study involving the intraperitoneal administration of SS resulted in a prolonged survival in the syngeneic mouse leukemia model L797 [166].

The human AML cell line, HL-60, exhibited a reduction in DNA, RNA, and protein synthesis in the presence of selenium, as assessed by examining the uptake of [^3^H]-thymidine, [^3^H]-uridine, and [^3^H]-leucine, respectively [166]. The synthesis of DNA was reverted to normal in cells upon the removal of selenium from the media, indicating that the effects of selenium were attributed to an interference with DNA biosynthesis (e.g., DNA replication and transcription) rather than a DNA template damage [166]. Additionally, Wu et al. demonstrated that sodium selenite induced cell cycle arrest as well as cytotoxic and pro-apoptotic activities in HL-60 cells by regulating pathways related to c-Jun NH2-terminal kinase 1 (JNK1), p21, p27, and cyclin D1 [161]. Misra and co-workers showed that pharmacological concentrations of selenite-induced cytotoxic activities by targeting the PML/RARα oncoprotein in the human acute promyelocytic leukemia cell line with t (15:17) chromosomal translocation NB4 and in combination with all-*trans* retinoic acid facilitated the induced maturation of these cells [167]. Sodium selenite (0.6 μg/mL) was also active towards primary leukemia patient samples (AML and ALL), inducing a cytotoxic yet selective effect, wherein healthy cells such as normal bone marrow or peripheral blood cells were not affected [166]. A reduced cellular proliferation and an induced apoptosis in lymphoma cell lines and primary lymphoma cells were demonstrated in the presence of MSA and SDG [162]. These findings indicate the prospective therapeutic value of the clinical development of selenium compounds in leukemias and lymphomas.

### 4.2. Selenium Impacts AML in In Vitro and In Vivo Models

Mutational and expression-profiling studies can provide insights into the modified epigenetics or transcription pathways of AML patients to uncover key genes or pathways that could be targeted to inhibit leukemogenesis [168]. A bioinformatic analysis by Jing et. al., involving pan-cancer super enhancer profiling and CRISPR dropout screens, revealed that the selenoprotein synthesis pathway may have critical implications towards AML [168]. The data from The Cancer Genome Atlas (TCGA) indicated that the *SEPHS2* gene, which plays an important role in the selenoprotein synthesis pathway, is greatly upregulated in AML patients relative to healthy patients’ blood cells [168]. The CRISPR deletion of genes critical to the synthesis of selenoproteins, such as *SEPHS2*, *SEPSECS*, and *EEFSEC* in human AML cells (e.g., MOLM-13, Kasumi-1, THP-1, and patient-derived xenograft samples) and murine AML cells with an MLL-AF9 mutation exhibited a reduced proliferation and an enhanced production of ROS relative to healthy cord blood and myeloma cells (U266B1) [168]. Transplantation studies of these modified AML cells into secondary recipients slowed the disease progression, reduced leukemia burden, and prolonged the survival [168].

A biochemical analysis of CRISPR-modified AML cells by Jiang and co-workers suggested that the deficiency to produce antioxidants, such as *GPX1* and *GPX4*, disrupted the redox environment necessary for the viability of AML cells, as evidenced by the reduced antioxidant Gpx4 and increased DNA damage marker **γ**-H2AX protein expression levels [168]. This was further supported by the slightly improved cellular proliferation of these CRISPR modified AML cells in the presence of the widely employed antioxidant and stable aminoxyl radical species, 4-hydroxy-2,2,6,6-tetramethylpiperidin-1-oxyl (TEMPOL) [168]. The AML murine models that received a diet deficient in selenium experienced a prolonged survival, reduced Gpx4 protein expression, and enhanced ROS production versus the control subjects that were fed a selenium adequate diet [168]. These findings suggest that the disruption in the production or functionality of selenoproteins may be a promising step towards improving the poor prognosis of AML patients [168]. Further pre-clinical and clinical investigations and a retrospective examination of these studies would be necessary to fully understand the benefits and disadvantages of supplementing or reducing the selenium levels in cancer therapy.

An epigenetic analysis done by Khalkar and co-workers showed that selenite and MSA affect distinct gene sets in the human AML cell line K562 [169]. These selenium-based compounds impacted the pathways related to the response to oxygen and hypoxia, cell adhesion, and glucocorticoid receptors, wherein a reduced cellular adhesion was observed among K562 and primary AML patient cells that were treated with MSA [169]. This suggests that selenocompounds may be able to weaken the interaction between leukemia and stromal cells in bone marrow tissues to allow chemotherapeutics to exert a greater cytotoxic effect on leukemia cells, thereby slowing the progression of AML [169].

Jin et al. demonstrated that polysaccharide nanotube-containing selenium nanoparticles induced potent cytotoxic activity in AML cells wherein the cellular proliferation was reduced, apoptosis was triggered, and cell cycle progression was arrested [170]. Further, the antioxidant activities were augmented in AML cells upon treatment with polysaccharide nanotube-containing selenium nanoparticles, as supported by the reduced protein expression of two oxidative stress related substrates, namely, c-Jun activation domain-binding protein 1 (Jab1) and thioredoxin 1 [45,170]. AML patient-derived xenograft cells derived from a hyperleukocytic AML-M5 patient with *NPM1* and *DNMT3A* mutations were intravenously injected administered the polysaccharide B-NSG mice and intraperitoneally administered the polysaccharide nanotube containing selenium nanoparticles (0.05 mg/kg) [170]. The in vivo therapeutic efficacy was observed, and the mice exhibited a slowed loss of body weight, prolonged survival, and a reduced population of human leukemia cells in the blood and bone marrow, indicating the reduced progression of the disease [170]. The significant anti-cancer activities imparted by these selenium nanoparticles in the AML cells and murine models supported the therapeutic promise of this agent in AML [170].

The treatment of the human AML cell lines, ML-2 and HL-60, with SLM resulted in a decreased cellular proliferation and induced a pro-apoptotic effect [171]. Immunocompromised mice that were subcutaneously injected with ML-2 or HL-60 luciferase expressing cells were then treated with either the vehicle, SLM, the standard AML chemotherapeutic cytarabine (Ara-C), or the combination of SLM and Ara-C. The in vivo efficacy of the single and combination treatment groups was comparable to the vehicle-treated control group. However, the anti-angiogenic and anti-tumor activities of the anti-VEGF antibody, bevacizumab (BV), with the addition of SLM, was augmented after 3 weeks of treatment in these human AML xenograft murine models [171]. The data from the bioluminescent imaging and tumor volume and weight measurements demonstrated the synergistic interactions between SLM and BV to reduce the disease progression in the HL-60 and ML-2 AML xenograft models. For instance, the mean tumor volume was further reduced by 13% in the HL-60-injected mice that were treated with the combination of SLM and BV relative to a single treatment with BV [171].

Annageldiyev et al. [172] observed the in vitro potency and in vivo efficacy imparted by phenybutyl isoselenocyanate (ISC-4) in human and mouse AML cells lines (e.g., MOLM-13, MV4-11, OCI-AML2, U937, and C1498) and human AML primary patient samples. In addition, ISC-4 exerted a selective cytotoxic and pro-apoptotic effect in human AML cell lines, primary human AML cells, and primary human AML leukemic stem cells versus cells from healthy patients, which were unaffected by the ISC-4 treatment. Synergistic effects were shown upon treating human AML cell line U937, primary human AML cells, and primary human AML stem cells with ISC-4 and Ara-C. The combination treatment significantly reduced the viability and augmented the apoptotic population in cancer cells relative to the single drug treatment [172]. In addition, ISC-4 has been shown to downregulate the PI3K/Akt pathway, resulting in the induction of apoptosis in melanomas as well as in prostate and colon cancers [173,174,175,176]. The PI3K/Akt signaling network is commonly upregulated in AML and is correlated with the poor prognosis of AML patients [172]. The anti-leukemic activity of ISC-4 was attributed to downregulating the PI3K/Akt signaling pathway and thereby activating caspase-regulated apoptotic signaling pathways (e.g., cleavage of caspases 9 and 3 and PARP).

**Figure 5:**
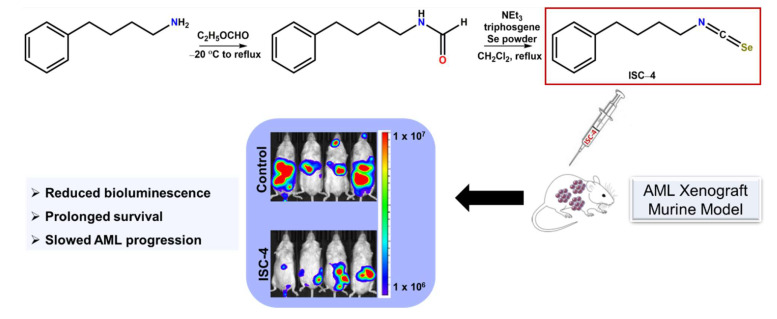
Synthesis of isoselenocyanate (ISC-4) and its efficacy in AML derived xenograft mouse models.

A significant reduction of disease progression was observed in syngeneic murine AML xenograft mice treated with ISC-4 (7 mg/kg; i.p.) versus the vehicle (Figure 5), as measured by bioluminescence imaging, flow cytometry analysis of leukemic cells in the liver, and an enhanced overall survival. The reduced bioluminescent signal on day 16 (Figure 5) effectively illustrated the diminished metastasis of cancer in ISC-4-treated mice relative to the vehicle-treated control group [172]. Immunocompromised NRG mice intravenously injected with the human AML cell line, U937, and subsequently intraperitoneally treated with ISC-4 (7.5 mg/kg) exhibited a decreased human CD45^+^ population in the bone marrow via flow cytometry versus the control group [172]. In this study, immunocompromised mice were also intraperitoneally administered either Ara-C (50 mg/kg) or a combination of both Ara-C and ISC-4. A comparable reduction in the human CD45^+^ populations of the bone marrow was shown in the mice treated with either ISC-4 or Ara-C relative to the vehicle-treated mice. Synergistic efficacy was observed upon treating the mice with a combination of ISC-4 and Ara-C (Figure 4), where a significant decrease in the human CD45^+^ population of the bone marrow tissue was seen relative to the control and single drug treatment groups. The synergism shown between ISC-4 with conventional AML chemotherapeutic Ara-C and the significant reduction in the leukemia burden in AML mouse models suggests the potential for ISC-4 to be clinically utilized in the treatment of AML [172].

### 4.3. Selenium Is Potent in Leukemia Stem Cells through In Vitro and In Vivo AML/CML Models

Leukemia stem cells (LSCs) are responsible for generating bulk leukemia cells that are correlated with the pathology of blood cancers, and relative to bulk leukemia cells, LSCs can induce leukemia in secondary recipients [177,178,179]. These LSCs are also related to the chemoresistance and relapse that are hallmarks of this disease [177,178,179]. Hematopoietic stem cells that express the BCR-ABL1 fusion protein from the 9:22 translocation can give rise to LSCs in chronic myeloid leukemia (CML) [177,180]. Conventional chemotherapy, such as the use of tyrosine kinase inhibitors, can inhibit the proliferation of bulk leukemia cells and allow for the remission of CML [177,178]. Standard agents are not active towards LSCs, and thus targeted therapies that can exert a cytotoxic effect on LSCs are necessary to prevent relapse [177,178]. Sodium selenite (Na_2_SeO_3_) has been shown to reduce the leukemia burden by stimulating p53 to induce apoptotic pathways [166,181,182]. Selenium-cystine administered to CML and AML patients, in the form of diselenodialanine, resulted in reduced total leukocyte and immature leukocyte counts [177,183].

Gandhi and co-workers investigated the role of selenium, in the form of inorganic selenite and organic methylseleninic acid, in the metabolic pathways in cancer stem-like cells (CSC) [177]. The authors observed that selenium, at supraphysiologic and nontoxic doses, induced selective ATM-p53-dependent apoptosis and higher intracellular oxidative stress (or levels of ROS) in chronic or acute myeloid leukemia stem cells among two established leukemia murine models and in AML or blast-crisis CML patient samples. However, these treatments spared hematopoietic stem cells. The blood from human patients diagnosed with AML or blast-crisis CML was examined and the CD34^+^CD38^-^CD123^+^ LSC population had an increased percentage of apoptotic cells upon treating the blood with lipid extracts from selenium-supplemented macrophages. It was also demonstrated that selenium increased the endogenous formation of the cyclooxygenase-based prostaglandins (CyPGs), Δ-12 prostaglandin J_2_ (Δ^12^-PGJ_2_) and 15-deoxy-Δ12,14-prostaglandin J_2_ (15d-PGJ_2_), suggesting that the apoptotic properties of selenium are in part associated with the endogenous eicosanoids [177].

Serial transplantation assays in mice with BCR–ABL-expressing CSCs indicated that selenium levels are important for the survival of CSCs [177]. The properties of selenium in primary human and murine leukemia stem cells were linked to the arachidonic acid metabolic pathway. This was further supported by the negative impact on selenium and its ability to stimulate p53 and induce apoptosis in leukemia CSCs in the presence of nonsteroidal anti-inflammatory agents (NSAIDs, e.g., indomethacin) and NADPH oxidase inhibitors. The inhibited cellular proliferation in LSCs by selenium, which was hindered by the presence of NSAIDs, indicates that the apoptosis triggered by selenium was in part correlated with augmented oxidative stress facilitated by NADPH oxidases such as Nox1. The selectivity imparted by selenium that is active in LSCs versus healthy hematopoietic cells provides support to the notion that altered intracellular ROS levels are important in the viability of LSCs [177]. A similar study by Finch et al. indicated that the selective cytotoxic effect in leukemia stem cells, upon providing selenium supplemented diets to CML bearing mice, is linked to the endogenous cyclopentenone prostaglandins production pathway by triggering the nuclear hormone receptor known as peroxisome proliferator-activated receptor **γ** (PPAR**γ**) [184]. These results suggest the promise of selenium supplementation as a CML adjunct therapy [177]. The efficacy of this treatment will depend on the selenoprotein expression and the regulation of the arachidonic acid metabolic pathway to drive the production of cyclooxygenase-based prostaglandins, thereby selectively inducing apoptosis in leukemia stem cells. Gandhi et al. demonstrates the significance of redox-dependent processes towards impacting the viability of leukemia stem cells [177].

### 4.4. Selenium Is Therapeutically Valuable in Patients with Hematological Malignancies

Multiple reports have demonstrated a relationship between low serum selenium levels and various hematological malignancies or solid tumor type cancers [13,14,185,186,187,188]. Hadjibabaie and co-workers found that twenty-two adult patients with hematological malignancies (Hodgkin disease, ALL, AML, CML, and non-Hodgkin’s lymphoma) had low mean selenium serum levels (19.91 μg/L, from 12.00 to 62.00 μg/L) prior to bone marrow transplantation (BMT) [117]. Aggressive non-Hodgkin’s lymphoma patients were studied, and it was found that the serum selenium concentration upon diagnosis can be independently related to their prognosis (e.g., treatment response and survival) [189]. Reduced serum selenium levels in patients with hematological malignancies have been correlated with poorer chemotherapy dose delivery, tumor response, and survival [188,189]. A lower cancer incidence was shown upon providing selenium compounds to selenium-deficient patients [18,190].

Selenium has been established to be a therapeutically beneficial nutritional supplement for cancer patients, and selenium supplements are often prescribed to patients with hematological malignancies [190,191]. Morel et al. found that a selenium-supplemented diet consumed by survivors of childhood acute lymphoblastic leukemia protected these patients from low HDL-C and cardiometabolic complications after cancer treatment [192]. It was also shown by Rocha et al. that pediatric leukemia or lymphoma patients that received dietary supplementation with selenium had lower neutropenic incidences and diminished IgG and IgA levels [193]. However, clinical studies have utilized various doses and different selenium compounds, primarily SS and SLM, indicating that further investigations are necessary to establish the most efficacious dose and the pharmacodynamic endpoints of these clinically-employed compounds [190]. Chronic lymphocytic leukemia (CLL) and patients with solid malignancies, as part of a phase I clinical study, received 400 μg of elemental selenium as either SS, MSC, or SLM for eight weeks to establish the safety, tolerability, and pharmacokinetic profile of these agents [69]. These compounds did not induce significant toxicities or genotoxicity [69]. Patients were able to sufficiently tolerate these compounds, as these elemental selenium analogues increased the total plasma selenium AUC (more Se with SLM) and slightly decreased the lymphocyte counts. Therefore, this study demonstrated the clinical utility of selenium-based compounds and provided a promising potential advancement of standard cancer treatments [69].

Preclinical and clinical studies have demonstrated the anticancer activities exhibited by selenocompounds and their potential to work synergistically with current cancer therapies [69,190]. Researchers have reported that the incorporation of selenium compounds into various conventional chemotherapy regimens, hormonal drugs, or radiation treatments enhances their therapeutic properties while diminishing some of their toxicities [93,194,195]. A combination of selenium with chemotherapy has been found to improve overall survival rates and to be an efficient immunomodulator [158,196,197]. Sodium selenite synergistically interacted with standard chemotherapy and exerted an apoptotic effect on lymphoma cells in non-Hodgkin’s lymphoma (NHL) patients [158]. Patients that received chemotherapy and adjuvant sodium selenite (0.2 mg/kg/day) for seven days presented increased apoptosis in lymphoma cells, as measured by flow cytometry, relative to the subjects that received only conventional chemotherapy (78.9 ± 13.3% versus 58.9 ± 18.9%, *p* < 0.05) [158]. Further, chemotherapy supplemented with sodium selenite resulted in a significant reduction of cervical and axillary lymphadenopathy, spleen size, and the percentage of bone marrow infiltration [158]. There was no diminished cardiac ejection fraction in the NHL patients that received selenium and chemotherapy, suggesting the cardioprotective properties of selenium [158]. This study indicates the potential for selenium to improve the prognosis of NHL patients [158]. Clinical studies have reported decreased chemotherapeutic associated toxicities in patients with hematological malignancies upon supplementing their treatment with selenium [64,158,190,197]. Larger clinical studies are needed to establish the most effective doses of selenocompounds and to extensively study the long-term therapeutic effects, toxicities, and potential secondary malignancies imparted by selenium to advance conventional cancer therapy in patients with hematological malignancies [65,190].

## 5. Selenium Alleviates Adverse Effects Associated with Radiotherapy or Chemotherapy

Radiotherapy is an effective cancer treatment that is employed to treat about 50% of patients diagnosed with cancer, and similar to chemotherapy, it can be associated with harmful side effects (e.g., normal tissue injury) [198,199]. The production of ROS from ionizing radiation contributes to the oxidative stress in cells that may result in negative protein modifications and adversely impact DNA, RNA, and cell membranes, leading to cell death and tissue damage [40,199]. The antioxidative activities of selenoproteins that allow them to reduce the formation of ROS brought on by radiation suggest the potential of selenium to improve the quality of life associated with radiotherapy [40,200]. The treatment of C6 rat glioma cells with selenite and irradiation demonstrated that selenium imparted a radiosensitizing effect, which resulted in the reduced plating efficiency and survival in glioma versus control cells [195]. Lobb and co-workers reported that low doses of MSA alone, or in combination with cytotoxic chemotherapy or gamma radiation, induced cytotoxic activities in malignant human mononuclear blood cells but spared the peripheral blood mononuclear cells (PBMCs) of healthy blood donors from cancer therapy-associated toxicity [201]. Supplementing standard cancer therapies with MSA also augmented the potency in malignant cells [201]. Greater cytotoxic activity was observed upon the treatment of malignant cells with higher MSA concentrations in combination with chemotherapy or radiation; however, these concentrations were toxic to normal cells and enhanced the cytotoxicity of the radiation [201]. This study suggested that the incorporation of selenium with chemotherapy or radiotherapy could offer a promising solution to improve the therapeutic properties of conventional cancer therapy and overcome the associated toxicity [201].

Clinical studies have shown that the supplementation of radiotherapy with selenium (300–500 μg/day over 10 days to 6 months) was well tolerated in patients with various cancers with no additional toxicity, where the blood selenium levels were augmented, the side effects associated with radiotherapy were decreased, and the efficacy of the radiotherapy was not compromised [40,65,202]. In a phase 3 clinical trial, selenium-deficient cervical and uterine cancer patients that orally supplemented adjuvant radiotherapy with 300–500 μg SS experienced higher blood selenium levels and a diminished number of episodes and severity of radiation-induced diarrhea, without compromising the long-term survival and therapeutic response imparted by radiation [65,203]. The quality of life was also noted among patients receiving chemotherapy supplemented with selenium for leukemia, lymphoma, or solid tumors, where fatigue and nausea as well physical, kidney, and liver functions were improved relative to the beginning of the study or compared to patients that received only chemotherapy [204]. Investigations that further establish and optimize the synergistic effects from supplementing standard cancer therapy with selenium may lead to improved prognoses and well-being in cancer patients.

Oncologists strategically utilize radiation therapy or chemotherapy to eliminate tumor cells aggressively and selectively versus healthy cells before potential tumor metastasis [205,206]. The combination therapy with chemo-radiation remains the standard of care for the management of several cancers despite demonstrating significant and often dose-limiting toxicity [207,208,209]. In vivo studies have demonstrated the promise of utilizing radiation-sensitive nanocarriers to deliver chemotherapeutics directly to tumor tissues [207,210,211]. The main drawback of this drug delivery system is the high radiation dose needed to release the drugs, which could present toxic effects to healthy tissues [207,212]. Diselenide-based nanocarriers have been shown to be biodegradable and radiosensitizing nanoparticles that are sensitive to ROS, resulting in the release of therapeutic agents and a nanocarrier breakdown into end products with beneficial antitumor and immunomodulatory properties [207,213,214,215].

**Figure 6:**
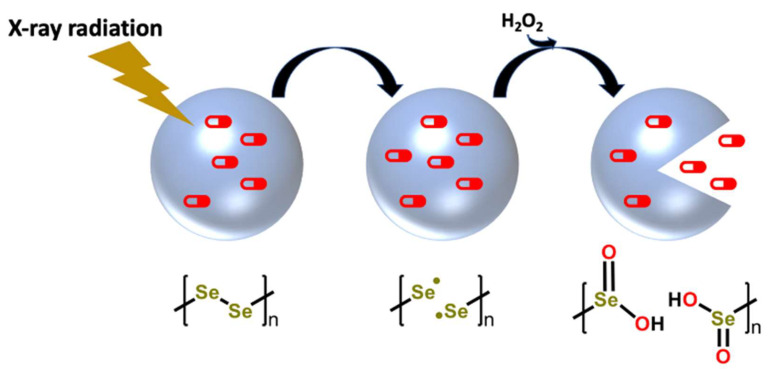
Diselenide nanocarrier-based drug delivery systems have been developed to selectively release chemotherapeutics in tumor tissues.

Zhang et al. [207] designed a drug delivery system in which diselenide block copolymers act as a nanocarrier to release the desired cancer treatment upon receiving a radiation dose of 2 gray (Gy) (Figure 6). The diselenium-derived polymer that makes up the nanocarrier (Figure 6) interacts with the X-ray dose and the high amounts of ROS commonly present in the tumor microenvironment to break apart and release anticancer agents [207,216,217,218,219]. Mechanistic insights were gained by nuclear magnetic resonance and density functional theory studies and suggest that the diselenium bond of the block copolymers is homolytically cleaved in the presence of X-ray radiation to produce selenyl radical species [207]. The high amounts of ROS (e.g., H_2_O_2_) present in various types of cancers results in its oxidization to a selenium-derived acid product and the release of enclosed chemotherapeutics [207]. Studies with 4T1 breast cancer tumor-bearing mouse models successfully demonstrated the delivery of anticancer agents in these diselenide X-ray-responsive nanocarriers to the tumor site with efficacious effects (reduced tumor volume/weight) and no additional toxicity [207]. The further development of diselenide nanocarrier drug-delivery systems may allow for a promising avenue leading to the reduction of the negative side effects of conventional cancer therapy that improve or at least do not compromise its efficacy [207]. Recently, an amorphous form of Selenium nanoparticles (α-Se NPs) synthesized using electron beam and stabilized within room-temperature ionic liquids (RTILs) as a host matrix provided great control over the size of the nanoparticles. In addition, the in vitro findings demonstrated that α-Se NPs exhibited the selective killing of A549 lung cancer cells over normal INT407 control cells [220]. Similarly, Wan et al., synthesized a porphyrin-containing covalent organic framework (COF) as a substrate for accommodating Se NPs, thus preventing the aggregation of Se NPs and enhance the stability. The combination of Se NP-mediated therapy with photodynamic therapy (PDT) greatly achieved long-acting and effective anticancer effects [221]. So, the futuristic studies exploring Se-based therapy alone or in conjunction with other therapeutics for the effective treatment of cancers are highly encouraged. Multiple reports have demonstrated the therapeutic potential of selenium nanoparticles in a wide range of biological processes and the exploitation of these properties may improve treatments for various diseases [124,170,194,222,223,224,225,226].

## 6. Conclusions

Selenium supplementation, via the addition of selenium to diets or the use of selenocompounds as drugs, can impart chemopreventitive and chemotherapeutic properties in various cancers. Different forms of selenium have been shown to impart their efficacy by regulating an array of biochemical pathways to induce cell death (e.g., apoptosis, cell cycle arrest, autophagy, etc.), affect gene expression or signaling networks relating to angiogenesis or tumor cell migration and invasion, or modulate the replicative properties of tumor cells or DNA repair/damage processes at different points of the cell cycle. The efficacy of standard-of-care cancer therapies, such as chemotherapy and radiotherapy, can also be improved, and the associated toxicities could be reduced with the addition of selenium. These findings have been substantiated in cell lines, animal models, and patients. Advances in cancer therapy will involve designing selenium-based compounds that are more effective and selective towards malignant cancer cells, less toxic to healthy cells, or that further benefit conventional cancer therapy. Efforts will continue to establish the role of selenium in various cancers and gain related mechanistic insights to aid in the development of targeted cancer therapies.

## Figures and Tables

**Table 1:** List of selenium-based compounds with promising anticancer activity.

S. No.	Compound	Redox Property	Cytotoxicity Mechanism	References
1	Selenate	Proapoptotic, genotoxic	Activates protein phosphatase 2A, which inhibits various signaling cascades such as phosphatidylinositol 3-kinase (PI3K)/Akt pathway. Induces apoptosis, but at a relatively high concentration.	[95]
2	Selenite/Sodium selenite (SS)	Proapoptotic, prooxidative, genotoxic, inhibits cell proliferation	Activation of extracellular signal-regulated protein kinase (ERK) pathway. Inhibition of autophagy through PI3K/Akt pathway	[96,97]
3	Selenocysteine (SeCys)	Antioxidant	Induces apoptosis through the cell cycle arrest, and oxidative damage. Paraptotic-like effect mediated by ER stress.	[98,99]
4	Selenomethionine (SeMet)	Proapoptotic, proliferation inhibition	Pro-apoptotic effects in several cancer cell lines. Activation of p53-dependent proteins. Non-toxic and non-genotoxic.	[1,85]
5	Methylselenocysteine (MSC)	Proapoptotic, anti-angiogenic, proliferation inhibition	Anti-cancer effects in various cell lines, including promyelocytic leukemia. ER stress and mitochondrial dysfunction/signaling	[83,100]
6	Methylselenic acid (MSA)	Pro-apoptotic, anti-inflammatory, pro-oxidant, anti-angiogenic	Induces cytotoxicity through DNA damage. Regulation of PI3k/Akt, *ERK1/2*, and p38 pathways	[77,101]
7	Selenodiglutathione (SDG)	Antioxidant, pro-apoptotic	Induction of apoptosis through ROS and oxidative damage.	[102]
8	Methylselenol	Pro-apoptotic; inhibits cell growth	Inhibition of the *ERK1/2* pathway activation and c-Myc expression. Induces cell cycle arrest.	[103]
9	Ebselen	Anti-inflammatory, antioxidant, protects against oxidative stress as well as DNA damage	As an antioxidant, Ebselen induces apoptosis through many pathways. Induces ROS generation and oxidative damage	[104]
10	Ethaselen	Proliferation suppression, synergistically effective with cisplatin against resistant leukemic cells	Induces ROS and apoptosis by TrxR inhibition	[105,106]
11	Dimethyl diselenide	Antioxidant	Induces NADPH quinone oxidoreductase	[107]
12	Zidovudine derivatives	Pro-apoptotic	Induced apoptosis through the mitochondrial pathway	[108]
13	Phenylindolyl Ketone Derivative	Pro-apoptotic	Induced apoptosis through cell cycle arrest and inhibition of tubulin polymerization.	[109]
14	Combretastatin 4-A analog	Inhibit tubulin polymerization	Inhibition of cell growth.	[110]
15	Diphenyl diselenide	Antioxidant, inhibitor of nociception	Protective against genotoxic substances. Induces apoptosis through oxidative damage.	[111,112]
16	Selol	Cytotoxic effects, inhibits proliferation, apoptotic	Induced apoptosis in resistant cancer cell lines including leukemia through oxidative damage.	[113]

## Data Availability

The data is available from the authors upon request.
